# Short-term and long-term effects of a social network intervention on friendships among university students

**DOI:** 10.1038/s41598-020-59594-z

**Published:** 2020-02-19

**Authors:** Zsófia Boda, Timon Elmer, András Vörös, Christoph Stadtfeld

**Affiliations:** 1 Chair of Social Networks, Department of Humanities, Social and Political Sciences, ETH Zürich, Weinbergstrasse 109, Zürich, 8006 Switzerland; 2 Department of Social Statistics and Mitchell Centre for Social Network Analysis, University of Manchester, Oxford Road, Manchester, M13 9PL United Kingdom; 3 Institute of Education, University College London, 20 Bedford Way, London, WC1H 0AL United Kingdom

**Keywords:** Psychology, Human behaviour

## Abstract

Informal social relations, such as friendships, are crucial for the well-being and success of students at all levels of education. Network interventions can aim at providing contact opportunities in school settings to prevent the social isolation of individuals and facilitate integration between otherwise segregated social groups. We investigate the short-term and long-term effects of one specific network intervention in an undergraduate cohort freshly admitted to an engineering department (${\bf{N}}{\boldsymbol{=}}{\bf{226}}$). In this intervention, we randomly assigned students into small groups at an introduction event two months prior to their first day at university. The groups were designed to increase mixed-gender contact opportunities. Two months after the intervention, we find a higher rate of friendships, common friends, and mixed-gender friendships in pairs of students who were assigned to the same group than in pairs from different groups (short-term effects). These effects gradually diminish over the first academic year (long-term effects). Using stochastic actor-oriented models, we investigate the long-term trajectory of the intervention effects, while considering alternative network processes, such as reciprocity, transitivity, homophily, and popularity. The results suggest that even though the induced friendship ties are less stable than other friendships, they may serve as early seeds for complex social network processes. Our study shows that simple network interventions can have a pronounced short-term effect and indirect long-term effects on the evolution and structure of student communities.

## Introduction

Informal social relations, such as friendships, have a decisive impact on the success and well-being of individuals at every stage of their lives^1–5^. Friendships made during the years of education have an exceptional effect on students, which they may carry on to adulthood. From early primary school to university, peer relations have been linked to students’ psychological well-being^6^, health-related behavior^7,8^, bullying and negative ties^9,10^, peer perceptions^11,12^, academic subject preferences^13,14^, and academic success or failure^15,16^.

If friendships made at school are so influential, it is important to know how students find friends among their peers. It appears that chance plays a considerable role in this process. Many people have anecdotes about how they met good friends seemingly randomly on their first days at school or university. Since the 1950s, social scientists have been studying this phenomenon and they have confirmed through experimental research designs that randomized seating and housing of students, as well as joint course taking can have a strong impact on the development of friendships between them^17–19^. Individuals who were randomly chosen to interact with one another were also more likely to perceive each other as attractive^20^ and to develop shared group identities^21^. Spatial closeness has been shown to increase the likelihood of interaction^22,23^ and to facilitate familiarity and interpersonal attraction^24^.

These consistent positive findings give hope that educational policies could directly influence students’ social networks by setting the context for meeting opportunities and interactions during the emergence of a student community. Targeted interventions could facilitate the social integration of individuals who are at risk of remaining isolated or foster the creation of ties between social groups segregated by, for example, gender, race, ethnicity, social class, or socio-economic status. Contact with members of a different social group reduces out-group prejudice and increases the likelihood of cross-group friendships^25,26^. These, in turn, contribute to equal access to social capital, thereby improving the labor market success of members of disadvantaged groups^27–30^. Thus, network interventions that create early contact opportunities could become a powerful tool to overcome inequalities in the education system and beyond.

But are students’ social networks that easy to influence? Recent advances in dynamic social network research show that friendship networks in educational contexts are highly structured^31,32^. In fact, certain patterns of friendship evolution almost seem universal. *Homophily*, the tendency to befriend similar others, is prevalent along most socio-demographic characteristics^33^ and could thus counteract the effects of interventions that aim at building bridges between social groups. Despite the decreasing importance of gender homophily in adolescence and adulthood^34,35^, mixed-gender friendships are still difficult to form and maintain in some settings^36^, even when they are promoted by institutional means^37^. *Transitivity*, the tendency to embed friendship relations in triadic structures^38,39^, has been argued to amplify the effect of homophily: if friends are more likely to be similar, connecting to friends of friends can further increase the level of network segregation, through the emergence of subgroups or clusters^40–43^. Other mechanisms can also alter friendship networks through time and thereby dilute effects of interventions. Important examples are *reciprocity*, the tendency to develop mutually tied friendship dyads or dissolve one-sided relations^44^, and *popularity* (or preferential attachment), the tendency to become friends with those who have many friends already^45,46^. Reasons for those network processes can be found in human motivations to form social ties, such as the need for belonging^47^, safety^48^, status^49^, or effectiveness^50^.

It is important to realize, however, that most network processes, like reciprocity, transitivity, and popularity, are unable to explain how initial ties appear in a community. This is because these processes merely capture how the presence of some social ties leads to the formation of others. For example, transitivity explains how two indirectly connected individuals will be more likely to become friends themselves. (Note that this is not true for homophily, in which case individuals are attracted to similar others independent of ties in the network.) Early friendship ties, which may partly be formed due to network interventions, could be crucial as they provide the basis for network evolution that unfolds later. However, their effect is not trivial. Intergroup contact theory highlights that contact opportunities may only foster positive attitudes and social ties in contexts where certain conditions are present^51,52^, although weak positive effects have been found in other cases too^29^. Therefore, to understand the full impact of early ties on social integration, we must study the effects of network interventions over a longer time period and relate them to structural processes of network formation.

In this study, we investigate the short-term and long-term effects of randomized first contact opportunities on friendship networks in an emerging community of first-year undergraduate students (N = 226) at an engineering department of a Swiss university [The Swiss StudentLife Study,^16^]. Students moved to the university from different parts of Switzerland and from all around the world and they had had little prior familiarity with each other before (0.38% of all dyads indicated to have known each other prior to week one). By the end of the academic year, they developed a dense social network, calling on average 4.2 of their fellow students a friend. By this time, 61% of respondents also reported that one of their best friends was among their peers at university, and 78% stated that they had a friend in the cohort whom they could turn to for emotional or study-related support. In our network intervention, we randomly assigned students from our sample to small groups at an introduction event before the start of their studies. We expected that those who were assigned to the same group would be more likely to be friends later. Moreover, we expected that pairs in the same group would have more friends in common, and that mixed-gender pairs would be overrepresented within the groups over the following months.

We specifically consider friendship relations between students of different gender (mixed-gender friendships). The 81 (36%) female students in the sample represent a potentially disadvantaged social group in this male-dominated engineering department. Educational research has argued that minority students, and females in STEM education in particular, may suffer from a lack of social integration which can negatively affect their well-being and academic performance^53,54^. Although many interventions are designed to attract and keep women in STEM study subjects [e.g., see^55^], women are still more likely to drop out from STEM careers than men^14,56,57^. It is suggested that cultural, cognitive, and behavioral barriers between male and female students could contribute to this^58,59^. Fostering mixed-gender interaction could reduce those barriers, as research on school children indicates^60,61^. Gender-segregated classes are argued to increase gender stereotyping, which can have negative effects on female performance in STEM^62^. In the context of this study, a network intervention could help female students to integrate better into the student community and thus reduce their likelihood of dropping out of the study program^16^.

### Combining a field experiment with an observational design

We use a combination of experimental design and social network panel surveys to assess the impact of meeting opportunities and early friendship ties on social integration in the student community. As part of our data collection, we conducted a field experiment and manipulated contact opportunities of roughly half of the students in the sample (${\rm{N}}=99$) who participated in either one of two *Student Introduction Days (SIDs)*. These events took place about two and three months before the academic year started. Students were randomly assigned to small groups (SID groups) to spend several hours together. The randomization happened under two conditions. First, all groups consisted only of students from one of the four study programs at the department. In the full sample, 97, 39, 59 and 31 students were following the different programs: those in the same study program shared most of their classes during the year, but the whole cohort also attended several classes together. Second, each group approximately reflected the gender composition of the department to increase mixed-gender interaction opportunities. Reflecting the exact gender composition of the department in each SID group would result in the highest possible number of mixed-gender pairs within SID groups. The randomization algorithm (see Methods section) did not create "optimal” outcomes in terms of maximizing mixed-gender pairs, but generated "sufficiently diverse” groups following a well-defined criterion.

While all students participating at the SID were part of the intervention, we consider the experimental treatment to be received by *pairs of students*. The experimental group thus consists of all pairs of students who were sorted into the same SID group, while our control group is all pairs of students who were not in the same group, but could have been, considering the constraints about study programs and gender composition (see Methods section). Since participation at the SID was not random but happened based on self-selection, in our main analysis we do not compare students who attended the events to those who did not; instead, we focus on comparing the effect of being in the same group to being in different groups at the SID. As the likelihood of friendships in treatment and control pairs is not independent (i.e. inducing ties with some peers may reduce the chance of forming ties with others), our setup can be deemed as quasi-experimental. In these small groups, the students experienced their first contacts with others from the same cohort.

**Figure 1:**
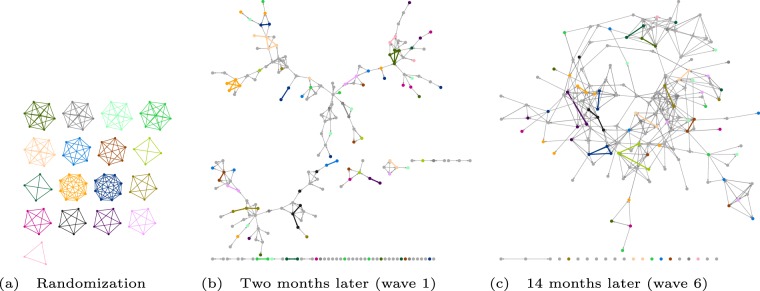
Social networks of the cohort at three time points. Panel (a) shows the randomized groups at the Student Introduction Days (SID groups) in which 99 students participated. Panel (b) shows the friendship network 2 months later, in the first week at university. Friendship ties that coincide with SID groups are highlighted in the respective color, others are shown in gray. Panel (c) shows the friendship network after the end of the academic year, 14 months after the randomization (6th data wave). Similar visualizations of waves 2–5 are provided in the Supplementary Information.

  Figure 1 presents the SID groups and the evolution of the friendship network of students over the first academic year. The result of the random assignment of students to groups at the two SIDs is shown in panel a of Fig. 1. We evaluate the short-term effects of the intervention in the first week of the academic year, two months after the randomization took place. Between the SIDs and the start of the academic year, there were no other organized events for the students to meet. The self-reported friendship ties between students at this time are shown in panel b of Fig. 1. We refer to these friendship ties in the first week as "early” friendships to note that they may be weaker and of qualitatively different nature than friendship relations reported later. The long-term effects are tested at multiple time points throughout the academic year, 3 to 14 months after the intervention. During this period, self-reported social network data were collected in six surveys, providing the opportunity to evaluate the persistence of the effects of the randomized field experiment. The friendship network from the sixth survey, conducted after the end of the first academic year, is shown in panel c of Fig. 1.

## Results

### Short-term effects: The creation of early friendship ties

We test whether students who had been assigned to the same SID group were more likely to be friends, to have common friends, and to have mixed-gender friendships in the first week of the academic year – two months after the network intervention. For this, we use the first wave of the friendship network survey data. At that time, 15% of all friendship ties were between individuals who were members of the same SID group. Overall, the network was still quite sparse, with a density of 0.8% (proportion of realized friendships within all possible ones).

The randomized assignment of students to SID groups followed a straightforward algorithm that is described in the Methods section. It ensured that SID groups consisted of students from the same study program and had a gender composition similar to that of the whole department (36% female). 17 groups were formed with sizes ranging from three to eight students. Thereby, 256 pairs of students had a shared group membership. These randomly formed pairs are compared to alternative pairings that could have been realized with the same randomization algorithm. We note that not each pair was possible (e.g., students from the same program participating on different days) or had the same probability, given the constraints on mixed-gender composition and same study program, as noted above. However, within these constraints we can carry out a comparison of pairs of students treated by the intervention (as they were assigned to the same SID groups) and pairs which were present at the SID but not treated (as they were assigned to different SID groups).

Given the very large number of possible randomizations, we sample 1,000 alternative SID group assignments and compare within-group statistics in these hypothetical samples to the empirically observed statistics (see the Methods section for details). The intuition behind this test is the following. If the randomization had no effect, we would expect that people who were in the same SID group have, for example, the same chance to be friends as people who were not in the same group but could have been assigned to one with the same probability. If the empirically observed statistic is higher than this expectation in a sufficiently large number of cases (95% if a significance threshold of $\alpha =0.05$ is considered), we can conclude that there is evidence that the network intervention had a positive effect on the formation of friendship ties.

**Figure 2:**
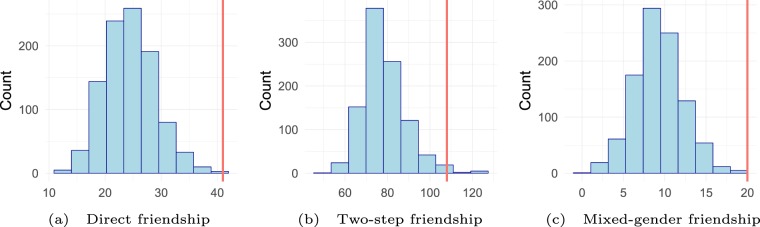
Short-term effects of the network intervention. Statistics are calculated with data from the first wave in the first week of the academic year. The distribution of statistics is shown under alternative randomizations of SID groups. The actually observed value is indicated by the red line and is significantly higher than expected for each statistic (p-values in panels a-c: $$ < 0.001$$, $$ < 0.05$$, $$ < 0.001$$).

The results of this comparison are shown in Fig. 2. The sample distribution of alternative randomizations is indicated by histograms, the empirically observed value by a red line. Panel a shows that 41 friendship relations were reported in the first week of the academic year between individuals who were members of the same SID group. On average, 24.5 friendship relations were found within groups under alternative SID groupings. A value equal to 41 friendship ties was obtained only in 1 of these 1,000 draws. From this we derive a non-parametric p-value of 0.001, providing strong evidence that the intervention had an effect on the formation of early friendship ties.

Panel b indicates a similarly pronounced effect for having common friends, which is operationalized as the number of two-step friendships (two-paths) in the friendship network between SID group members (see the Methods section for details). 108 two-step friendships were reported between members of the same group, on average 78.5 two-step friendships were found under alternative randomizations. The non-parametric p-value is 0.016 (6 randomizations were equal to, 10 larger than 108), indicating that the intervention has an effect on the network distances between individuals, even when they were not directly connected.

Panel c shows the number of mixed-gender friendship relations within SID groups. 20 mixed-gender ties were reported within the actual groups. In the alternative groupings, only 9.3 mixed-gender ties were observed on average. The non-parametric p-value is 0.000 under the 1,000 randomizations (no alternative randomizations with 20 or more mixed-gender ties), providing strong evidence that many early mixed-gender friendship ties are created through contact opportunities provided by the network intervention.

### Long-term effects: Persistence of the network intervention

We investigate how the intervention effects develop in the long term, using social network survey data collected six times during the academic year. Panel c of Fig. 1 shows the final friendship network in wave 6 (14 months after the randomization) as an example. Network plots from the other waves are provided in Fig. 1 of the Supplementary Information. The friendship network in wave 6 is clearly denser than in wave 1 (Panel b), indicating the emergence of a student community. In comparison to the overall network growth, it is noteworthy that the number of SID-group friendship ties did not further increase but decreased over time. The number of reported friendship ties between SID group members decreased from 41 in wave 1 to 19 in wave 6. This difference can be partly attributed to the dropout of some students (for 11 of those friendships, at least one person was not part of the cohort anymore) and a lower participation rate in the survey (63% in wave 6 as compared to 79% in wave 1).

**Figure 3:**
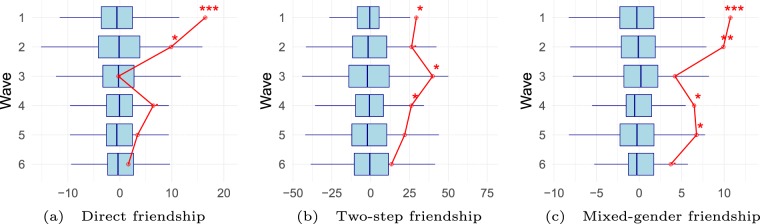
Long-term effects of the network intervention. The data stem from six data collection waves throughout the academic year. Variable distributions are generated from alternative randomizations of SID groups and are centered to facilitate readability. The red line shows the empirically observed values. Significance is indicated by ***$p < 0.001$, **$p < 0.01$, *$p < 0.05$, ^.^:$p < 0.1$.

  Figure 3 shows the distribution of statistics under 1,000 alternative SID group assignments at each survey wave. We calculate the same statistics as before (direct friendships, two-step friendships, mixed-gender friendships) but now evaluate their trend throughout the academic year. The statistics are presented in a similar way as in Fig. 2. The histograms are replaced with boxplots that show the first and third quartile of the distribution as well as the median. The red lines indicate the empirical values of the statistics that were centered for easier readability. The first row in each panel of Fig. 3 corresponds to the distributions reported in Fig. 2.

We find evidence that the randomization matters in later waves as well. On direct friendship, we still find a significant effect in wave 2, three months after the intervention and one month into the academic year. 44 friendship ties are observed between group members (the corresponding p-value is 0.048). From wave 3 onwards (five months after the intervention, three months into the academic year), fewer friendship ties between SID group members are reported (range 19–30), which are not significantly different from the number of friendship ties between non-group members who could have been in the same SID group. In wave 3, the empirical value equals the median of the distribution, in wave 4-6 it is higher. The non-significant p-values in waves 3-6 are 0.542, 0.084, 0.235 and 0.349.

Evidence for a two-step friendship effect – the tendency to have common friends with others from the same SID group – is found in waves 3 and 4 (p-values: 0.026 and 0.044, respectively), after being present in wave 1 (p-value: 0.016). The empirical values range between 108 two-step friendships (wave 1, randomized median: 78) and 212 (wave 3, randomized median: 170). The p-value in wave 2 is 0.066 and thus is not considered significant. We further find no significant evidence that individuals from the same group share more friends in waves 5 and 6 (9 and 14 months after the randomization; 7 and 12 months into the academic year; p-values of 0.112 and 0.217). In all six waves, the empirically observed number of two-step friendships is higher than the third quartile of the randomized distribution.

Evidence for a higher number of mixed-gender friendship ties between SID group members is found in waves 1, 2, 4 and 5 (p-values: 0.000, 0.003, 0.019, 0.012) but not in waves 3 and 6 (0.106, 0.066). The empirically observed number of mixed-gender friendship ties within groups ranges from 10 (wave 6, randomized median: 6) to 21 (wave 2, randomized median: 11). In all six waves, the empirically observed number of mixed-gender friendships is higher than the third quartile of the randomized distribution.

We conclude that the effect of the randomized SID group assignment on the number of friendships, two-step friendships, and mixed-gender friendships fades out in time. The effect on direct friendship is apparent until one month into the academic year, three months after the intervention. We find an effect on two-step friendship until seven months and an effect on mixed-gender friendship until nine months after the network intervention.

### Network effects: The evolution of friendship

**Figure 4:**
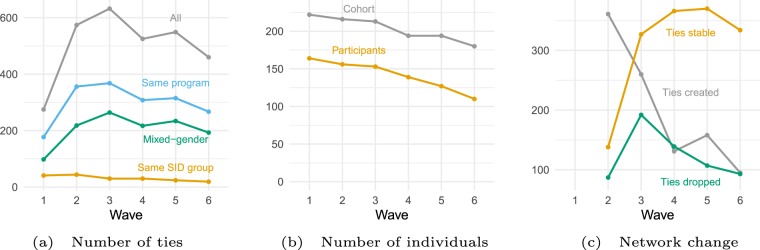
Network change descriptives.

From Fig. 1, it is apparent that the friendship network grew substantially through time. Figure 4 shows a number of statistics that describe this change. Panel a of Fig. 4 shows the number of friendship ties observed at each time point. The network in the first week was sparse, then the number of friendships rapidly increased, reaching their maximum in wave 3. However, based on panel B both the cohort size and the participation rate fell over time: taking into account missing information, the network is at its densest state in wave 6. The number of same-program and mixed-gender ties follow similar patterns as the overall number of ties. The number of friendship ties between SID group members is, at the same time, the highest in wave 2 (44).

To be able to better understand the role of randomness in friendship formation, we need to look at its diminishing effect in context. There was a lot of change in the network during the early phase of its evolution. Panel c of Fig. 4 shows that in the first months of network evolution, many new ties were created (the figure shows creation, deletion and stability of ties in comparison to the preceding wave); a little into the academic year, students also started dropping many of their existing friendships. Later, both of these processes slowed down, and the number of stable ties became higher. While the random grouping was one of the most important sources of familiarity between students at first, later other processes seem to have come into play, leading to a more stable network.

Which processes explain the formation and stability of these friendship ties? And how do SID ties affect these processes? We evaluate the social network processes in two stochastic actor-oriented models for network dynamics [SAOMs,^64^]. SAOMs express how likely it is for network changes to occur within certain structural configurations. The first model explains the formation of the network in wave 1. The second model expresses the network change *after* the formation of the early friendship ties by wave 1, *conditioning* the results on this first observation. We include parameters in the model to test whether reciprocity, transitivity, and self-reinforcing popularity matter in the process of network change. We further evaluate the effect of homophily regarding gender and study program. We note that the models further control for change rates period-wise, outdegree of actors, interaction between reciprocity and transitivity, degree distributions, individual gender effects, parental education, and participation at the SIDs. Details of the model specification are provided in Tables 1 and 2 of the Supplementary Information.

In both models, we further specifically test how the SID ties affect the evolution of the friendship network. We include variables to test for mechanisms for the overrepresentation of same SID group direct friendships, two-step friendships, and mixed-gender friendships. For direct friendship, we test whether individuals tend to form and maintain friendships with others from their SID group (in the second model conditional on those existing in the first week). For two-step friendship, we look at tendencies of forming and maintaining ties with friends of their same-SID peers, or with the same-SID peers of their friends. For mixed-gender friendship, we test whether they are more likely to be formed and maintained within a SID group than outside of it. This way, we can see how SID group membership directly and indirectly contributes to the creation and stability of friendship ties.

**Figure 5:**
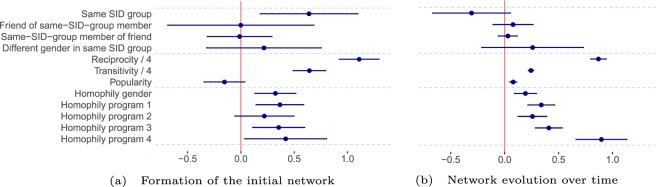
Processes of network change. Results from two stochastic actor-oriented models. Model A explains the formation of the network observed in wave 1. Model B expresses network change from wave 1 to 6 conditional on the observation in wave 1. The plot shows estimates and 95% confidence intervals of 9 core effects. The full model is presented in Tables 1 and 2 and section 3 of the Supplementary Information.

The SAOM results are presented in Fig. 5. The parameters in Fig. 5 can be interpreted as log-odds ratios to create and maintain ties in a particular network configuration. The first model (panel a) explains the network processes related to the first network observation in wave 1. Technically, it models the evolution from an empty network to the one in wave 1 (for details, see the Methods section). We find significant evidence that individuals tend to become friends with their same-SID peers (first row; p-value: 0.006). This result is in line with those presented earlier (Fig. 2, panel a), now additionally controlling of other network processes (i.e., the other effects in the model). We further find significant evidence for the presence of reciprocity and transitivity ($p < 0.001$ in both cases). We do not find evidence for clustering of friendship networks around SID ties (second and third row; p-values: 0.997, 0.937), for an over-representation of mixed-gender ties between SID members (row 4, p-value: 0.431) and for popularity mechanisms (p-value: 0.115). Although we do not find effects for clustering and gender mixing around SID ties in our dynamic model, they are, nevertheless, descriptive outcomes of the network at wave 1 (see Fig. 2).

The second model (panel b) explains the network change between wave 1 and wave 6 (thus conditioning on the observations of wave 1). We find weak evidence that individuals are less likely to maintain their existing SID friendships or to form new ones with those from the same SID group *beyond* the ties that they already reported in wave 1. This effect is marginally significant (p-value: 0.09). This mirrors the descriptive finding that the direct effect of the SID disappears over time and could suggest that the induced friendship ties are less stable than other friendship ties in the network. For the other three SID effects, we find positive, but also non-significant effects. We thus find no evidence that individuals maintain or form friendships with friends of same-SID peers or same-SID peers of friends over time (from wave 1 on; p-values: 0.411, 0.512, respectively). Similarly, we could not show that mixed-gender friendships would be maintained or newly formed between SID peers more than in the cohort in general (p-value: 0.282). At the same time, we find clear evidence that additional processes explain the network evolution. *Reciprocity*, *transitivity* and *popularity* increase the chance of a friendship to appear conditional on the presence of other ties in the network (all $p < 0.001$). We further find clear evidence for *homophily* regarding gender and regarding the four study programs (all $p < 0.001$). The results of the dynamic network models cannot be directly compared to the results reported in comparison with alternative SID randomizations (see Fig. 3, panels a–c) because the dynamic network model only models changes of ties and assumes constant processes of change over the time periods.

## Discussion

We presented experimental evidence that randomized early contact opportunities between first-year undergraduate students had a strong effect on the emergence of social ties between them. We proposed a straightforward network intervention in which students were randomly assigned to groups for a few hours on a student information day. They would typically see their fellow group members again only two months later on the first day of their undergraduate studies. Yet, when surveyed in the first week at university, students from the same intervention group were clearly more likely to report friendships and mixed-gender friendships, and they had a higher number of common friends. These findings contribute to a line of literature on social network interventions from sociology, social psychology, and social networks research [e.g.,^17,21,63^].

The network intervention had the strongest effect at the beginning of the academic year when students formed early friendship relations. Over the year, the network first grew, and then kept changing as individuals found new friends and dropped other friendships. These processes appeared to dilute the effect of the intervention. We found significant evidence for being friends with same group members until 3 months after the intervention, for having more shared friends with them for 7 months, and for having more mixed-gender friendship relations for 9 months, almost until the end of the academic year. While there is an abundance of theoretical arguments and empirical research on the role of chance encounters in the formation of positive social ties, the long-term effects of such interventions are rarely investigated. Our social network panel design enabled us to study these effects in more detail.

To further investigate the decreasing importance of early mixing over time, we modeled the social network longitudinally, starting with the very first state of friendships measured early on. We tested for mechanisms behind friendship formation and maintenance: whether those who were in the same group are more likely to become or stay friends, whether individuals find friends through those they were in the same group with, or whether mixed-gender friendships are more likely between those who shared the same group. We find evidence that students were more likely to create same-group ties at the beginning of the semester, controlling for a number alternative network processes, and weak evidence that these induced friendships ties were less stable over the academic year.

Even though randomly induced early friendships did not seem to be significantly different from other early friendship in their further evolution, they were over-represented among them at the beginning of the community formation. Thus, they may have still played an implicit role in the subsequent network evolution. This is because the evolution of the network was driven by various structural processes (for example, reciprocity, transitivity, and self-reinforcing popularity) that require the presence of some ties to operate. Early friendship ties, among which those induced by the network intervention are overrepresented, can thus serve as seeds in the emergence of complex social structures. Our study contributes to the literature on social network dynamics by showing how random early interactions and structural processes jointly affect the emergence and evolution of social networks. Future research could further aim at understanding the role of early social ties as seeds in the long-term network formation process. The statistical methods that we applied can in principle test such detailed mechanisms, but possibly require larger samples to avoid statistical power issues^65^. Higher response rates than those achieved in our study could also provide sufficient power, while also decreasing the risk of biased estimates due to systematic non-response.

Our randomization algorithm oversampled mixed-gender pairs in groups to facilitate the creation of social ties between female and male students. The importance of this is to be understood in the context of the engineering program that we studied. Managers of this study program are aware that female students (who are the minority in the community) tend to feel less socially integrated, which can negatively affect their well-being and academic success. Female students’ social isolation in male-dominated study programs (especially in STEM subjects) is an important research line in the social sciences, and it is considered a crucial explanation for the often higher female dropout from degrees in STEM subjects^53,54,66^. After our intervention, we observe more mixed-gender ties between those who were randomly sorted into groups together. However, one limitation of this study is that our research design did not permit to demonstrate whether the intervention itself increased the number of mixed-gender relations compared to an intervention in which no mixed-gender contacts were induced. In order to test experimentally if early contacts lead to higher mixed-gender integration or different attitudes, future studies could compare students who are randomly exposed to more peers of the opposite gender to students who are not. This could be done by constructing and comparing gender-homogenous and heterogenous groups. However, when taking such an approach researchers need to carefully consider research ethics as students might end up with different levels of gender integration in their networks which could potentially affect their well-being, gender norms, or academic opportunities.

At the same time, there are some indications in our study that the participation in the network intervention may have positively affected social integration between genders. In a non-experimental post-hoc analysis, we compare the 99 students who participated in the introduction events and who were therefore part of the network intervention with the 127 students who did not sign up for these events. We find that the proportion of mixed gender friendships is higher for SID participants through all data collection waves (see Fig. 2 in the Supplementary Information). Intervention participants further reported more direct friendship ties and two-step friendship ties than non-participants, indicating that the intervention might have affected the level of students’ social integration. Again, it is important to highlight that participation at the SID was not random (students chose to sign up) and thus conclusions based on the comparison of those who did and those who did not attend should be made with caution. Nevertheless, these descriptive findings call for future research on the effect of network interventions that aim to facilitate the social integration of students and foster between-group interactions. Such field experiments could connect to quasi-experimental findings of educational sociologists on the role of contact opportunities in the integration of different social groups^67^. Another limitation is that our findings may be specific to the educational, cultural, and temporal context of our empirical setting. To generalize our findings about the short-term and long-term effects of educational network interventions, additional studies in different contexts are required. In case it is possible to successfully induce more mixed-gender ties, future interventions could offer policy makers important tools to reduce cultural, cognitive, and behavioral barriers between male and female students. Thereby, they may contribute to the mitigation of gender stereotyping and performance gaps in STEM subjects^58,60–62^.

Chance matters in how we meet our friends. Everyday decisions by university teachers and managers on, for example, who to assign to study groups together or who is sharing the same mentor can have an effect on who becomes friends with whom. This implies that such decisions should be taken in a thoughtful manner, for example, by avoiding shortcuts like sorting students by last name and thereby inducing imbalanced groups in terms of ethnic background. To avoid implicit biases of that kind it seems advisable to randomize meeting opportunities of students if no specific goals in terms of social integration are pursued. If university managers and teachers want to foster social integration of potentially disadvantaged groups (like female students in a STEM program in our empirical case) they could potentially use such network intervention to increase the chance of between-group ties. The power of contact opportunities can be considered in educational strategies that aim at facilitating between-group contacts and mitigating the risk of social isolation, in an attempt to increase well-being and academic success of students^16^.

This article showed that network interventions can have a strong effect on the formation of early social ties. We illustrated that even small interventions, like a random group assignment at a student information day, can have effects that are visible for several months. The effects may diminish when individuals change their social networks through time, driven by structural processes that, for example, explain individual preferences for homophily, transitivity and popularity. We further demonstrated how the interplay between random meeting opportunities and structural network processes can be jointly investigated in one empirical setting, and that both aspects contribute to the emergence of social networks. Friendship ties are crucially important for students. They have been linked to diverse outcomes such as well-being, inter-group attitudes, and academic success. Network interventions in educational contexts can be powerful tools to facilitate early friendship ties between students. These ties may affect the perceived social integration of individuals, and serve as seeds for the emerging social structure.

## Methods

### Data

We analyze data from a group of freshly admitted undergraduate students at a Swiss university in the academic year 2016–17. Descriptive characteristics of the sample are presented in Fig. 4, and the timing and participation rates of the key data points are shown in Table 1. For the SID intervention, the relevant cohort is 99 students who were all part of the intervention. For the survey waves, the cohort size includes students enrolling in the four study programs at any given time. This ranges from 222 to 180. More details about the dataset can be found in section 2 of the Supplementary Information. The data we analyze here comes from two sources: from the network intervention at an early introduction event for students and from online surveys administered six times throughout the academic year.

**Table 1:** Timing of the data collection. Participation rates are calculated from students enrolled in the study program at the time. Due to delayed dropout the actual participation rate is expected to be higher. The first row reports the number of students who were part of the community at the beginning and the ratio of these students who participated in at least one survey.

Time	Type	Participation	Cohort size
June/July	SID intervention	100%	99
Mid-September	Wave 1	77%	222
Mid-October	Wave 2	72%	216
Mid-December	Wave 3	70%	213
Mid-March	Wave 4	70%	194
Late May	Wave 5	66%	194
Late August	Wave 6	61%	180
	All survey waves	86%	226

Randomized grouping of students took place at two university-organized one-day information events called *Student Introduction Days (SIDs)*. New students were invited to participate in one of two SIDs, the first one taking place in mid-June and the second one in early July 2016, two months before the start of the academic year. One SID took place about two months, the other about three months before the start of the academic year. When we speak of “two months” in the following, we refer to both SIDs. In total, 99 individuals attended one of the two SIDs (${N}_{1}=31$; ${N}_{2}=68$). Participation at the events was not random (e.g. students living in Switzerland were more likely to attend than students from abroad) and students could choose their preferred day of participation. Each SID started with a brief introductory lecture, after which students were distributed into small groups (SID groups) for further orientation: a tour through the university campus, a discussion round, and a meal. The group program was led by older students from the same study program who volunteered to act as mentors. The groups consisted of 5-9 students. Not all students who participated in the SIDs enrolled at university later (11 students dropped out before the start of the academic year). These cases are treated in our study as if the student had never been part of a group, affecting the group size (leading to a minimum group size of 3 and a maximum group size of 8; see Fig. 1). One condition was given by the context: only students from the same study program were assigned to the same group. As a second condition, we aimed at oversampling mixed-gender ties. The group sizes were also determined by the number of available tutors per major and the number of students from a specific study program who signed up for each of the two days. The algorithm used for the grouping is formally defined in Algorithm 1 below.

**Algorithm 1:**
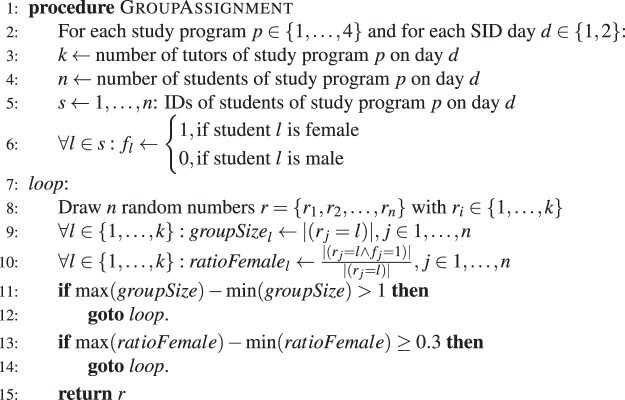
Group randomization.

It is noteworthy that the algorithm does not necessarily optimize the group gender ratio. It instead considers a threshold of a 30 percentage point difference to determine when a randomization was "sufficiently diverse”. The algorithm was implemented in Microsoft Excel using a random number generator for the SID randomization. The exact procedure was minuted. For the sampling of alternative randomizations, the same algorithm was implemented in R^68^ and used to generate 1,000 alternative group assignments. The gender composition threshold was chosen arbitrarily prior to the randomization, but used consistently throughout the process. Even though the threshold of 30 was a constant, we treat it as a variable in Fig. 3 of the Supplementary Information to illustrate that under the assumption that the randomization had been generated with thresholds of 25, 35 or 50 the experimental results are unchanged. The actual randomization has a difference of 29 percentage points between groups.

For collecting data about students’ friendships and individual attributes, six longer, detailed online surveys were administered during the year. The first survey was distributed in the first week of the academic year (September), the second one month later (October), the third another two months later (December). There were two additional questionnaires in the second half of the year: in March and June, and a sixth one at the end of August, right after the first-year exam students took. In the first wave, we invited all participants to a computer lab to fill out the questionnaire: this way, our trained research assistants were able to answer questions of participants. Most students filled out the survey in a computer lab. We still offered this option in later waves, but the vast majority of students opted for filling out the questionnaire on their own computer upon receiving the survey online link. Students were also incentivized by a monetary prize, in line with the university’s research policy.

### Measures

In each of the six surveys, friendship networks were measured with the following item: “Which of your fellow students would you consider a friend?”. Participants could nominate up to 20 of their fellow students by typing their names in designated text boxes. An auto-complete function suggested the full names of other students of the cohort when participants started typing in a text box. A friendship network from each survey was created using these nominations (1 = friendship, 0 = no friendship between each pair of students).

When studying the dynamics of friendship formation, it is important to take into account a number of individual characteristics that are known to influence friendship choice in similar contexts. Here we focus on five key individual variables that were assessed in the first wave of the survey. For capturing within-cohort differences, we include the study program (dummy variables for the four programs). For capturing more general demographic variables, we include gender (1 = female, 0 = male), age (expressed in years and months, calculated for September 2016), and country of origin (1 = Switzerland, 0 = other country). We also included the mother’s level of education (primary, secondary, or tertiary education) as a measure for socio-economic status.

### Analytical strategy

We study the effects of initial meeting opportunities on friendship formation in two steps. First, we show the effect of SID group assignment on friendship ties, two-step friendships and mixed-gender friendships. Second, we look at the network evolution over time using stochastic actor-oriented models (SAOMs).

In the first step, we present the number of friendship ties observed in each survey wave between students who were in the same SID group. We compare each observed number to a meaningful null distribution using a permutation test approach [see, e.g.^69^]: we calculate the same statistic on 1,000 hypothetical realizations of the SID group assignment that consider the conditions of the grouping procedure (SID participation, study program and gender). This way, we are able to statistically assess the relationship between being in the same SID group before the beginning of the year and friendships reported during the year. Significance of a finding is evaluated by calculating a p-value in a one-sided statistical test. This statistical procedure is known as “conditional uniform graph test”^70^. The baseline distribution that we condition on consists of the possible SID assignments and their respective probabilities, given constraints on group sizes and gender ratio.

Similarly, we look at the effect of shared SID group membership on two-step friendships and mixed-gender friendships. For the two-step friendship statistic, we count the number of common friends for each pair of individuals who belonged to the same SID group (regardless of whether the pair reported each other as friends or not). We consider someone a common friend when she is linked to both individuals of the pair, either nominating or being nominated by each of them as a friend. While the number of common friends is positively correlated with being direct friends, it also quantifies how strongly students are embedded in the same friendship groups and how much they are exposed to similar norms and information from peers. For the same-gender friendship analysis, we count the number of same-gender friendships between SID group members. In both cases, we compare the observed counts to null distributions of 1,000 hypothetical scenarios where the grouping of students is different but equally probable under the given randomization conditions.

In the second step, we apply stochastic actor-oriented models [SAOMs^64,71^]; to investigate patterns of the network evolution. SAOMs are statistical models developed for the dynamic analysis of social networks, when traditional regression-based methods cannot be applied due to the lack of independence of observations (i.e., social ties). SAOMs model changes in a network between two or more observed time points, estimating parameters for processes driving the evolution of social ties. SAOMs can also be understood as agent-based simulation models which are calibrated based on observations of real-world networks.

In SAOMs, network change is assumed to be driven by actors, who make decisions to create, maintain, or delete ties to other actors (hence the term “actor-oriented”^72^). Beyond individual characteristics of an actor, changes in her social ties also depend on her network neighborhood [^73^ p. 10]. The network neighborhood can be specified flexibly by a set of variables in the model. Estimation methods for SAOMs are implemented in the RSiena software package, which we use for the presented analyses. For more detailed descriptions of the model, see Snijders^64,71^ and Ripley *et al*.^73^.

The dependent variable in our SAOM analysis is friendship, which is explained by a set of independent variables. To capture the network structure, we include reciprocity (whether *i* is more likely to create or maintain a friendship towards *j* if *j* names *i* as a friend), transitivity (whether *i* is more likely to create or maintain a tie towards *j* if they have more friends *h* in common), and popularity (whether *i* is more likely to create or maintain a tie towards *j* if *j* is nominated by more other actors *h*). The mathematical definition of these variables can be found in^73^; the variable names used here are “reciprocity”, “transitivity”, and “popularity”, respectively. Beyond these, we include two additional variables capturing dynamics of degree distributions (“outdegree-related popularity” and “outdegree-related activity”), and one with the interaction between reciprocity and transitivity (“transitive reciprocated triplets”). Moreover, we include effects of the study program (same program for each of the four categories separately), gender (gender of the friendship sender, gender of the receiver, same gender of the sender and receiver – gender homophily), age (age of sender and receiver, similarity in age), country of origin (whether the sender, the receiver, or both are from Switzerland), and the mother’s education (education of the mother of the sender, the receiver, and their similarity). We also include SID participation (sender participated, receiver participated, both participated) and whether the sender and the receiver of the friendship tie were in the same SID group.

We fit two SAO models with the same model specification: one for the formation of the initial friendship network, and one for the network evolution between the six observations of friendship. For the former model, we assumed the starting point to be an empty network, and the endpoint to be the first observation of early ties. In the latter model, network evolution is simulated between every two subsequent waves, the first of the two serving as a starting point and second one as an endpoint.

## Research Ethics and Data availability

The study design and procedures were reviewed and approved by the institutional Ethics Commission of ETH Zürich (ethics approval number: 2016-N-27). All research was performed in accordance with relevant guidelines and regulations. Informed consent was obtained from all participants. Due to a privacy protection policy agreed with the Ethics Commission, the full data set is not released publicly. Anonymized parts of the dataset are made available to researchers upon request. The data access policy is detailed on the project website.

## Supplementary information

Supplementary Information.

## References

[1] Kawachi I, Berkman LF (2001). Social ties and mental health. J. Urban. Heal..

[2] Berkman LF, Glass T, Brissette I, Seeman TE (2000). From social integration to health: Durkheim in the new millennium. Soc. Sci. & Medicine.

[3] De la Haye K, Robins G, Mohr P, Wilson C (2010). Obesity-related behaviors in adolescent friendship networks. Soc. Networks.

[4] Granovetter MS (1973). The strength of weak ties. Am. J. Sociol..

[5] Coleman JS (1988). Social capital in the creation of human capital. Am. J. Sociol..

[6] van Zalk MHW, Kerr M, Branje SJ, Stattin H, Meeus WH (2010). It takes three: Selection, influence, and de-selection processes of depression in adolescent friendship networks. Dev. Psychol..

[7] Michell L, West P (1996). Peer pressure to smoke: the meaning depends on the method. Heal. Educ. Res..

[8] De La Haye K, Green HD, Kennedy DP, Pollard MS, Tucker JS (2013). Selection and influence mechanisms associated with marijuana initiation and use in adolescent friendship networks. Journal of Research on Adolescence.

[9] Huitsing G, Veenstra R (2012). Bullying in classrooms: Participant roles from a social network perspective. Aggressive behavior.

[10] Pál J, Stadtfeld C, Grow A, Takács K (2016). Status perceptions matter: Understanding disliking among adolescents. Journal of Research on Adolescence.

[11] Vörös A, Snijders TAB (2017). Cluster analysis of multiplex networks: Defining composite network measures. Social Networks.

[12] Boda Z (2018). Social influence on observed race. Sociological Science.

[13] Sacerdote B (2001). Peer effects with random assignment: Results for dartmouth roommates. The Quarterly journal of economics.

[14] Raabe, I. J., Boda, Z. & Stadtfeld, C. The social pipeline: how friend influence and peer exposure widen the stem gender gap. *Sociology of Education* 0038040718824095 (2019).

[15] Lomi A, Snijders TA, Steglich CE, Torló VJ (2011). Why are some more peer than others? Evidence from a longitudinal study of social networks and individual academic performance. Social Science Research.

[16] Stadtfeld, C., Vörös, A., Elmer, T., Boda, Z. & Raabe, I. J. Integration in emerging social networks explains academic failure and success. *Proceedings of the National Academy of Sciences* **116**, 792–797 (2019). 10.1073/pnas.1811388115 PMC6338824 30584099

[17] Byrne D, Buehler JA (1955). A note on the influence of propinquity upon acquaintanceships. The Journal of Abnormal and Social Psychology.

[18] Festinger, L., Schachter, S. & Back, K. *Social pressures in informal groups; a study of human factors in housing*. (Harper, 1950).

[19] Moreland RL, Beach SR (1992). Exposure effects in the classroom: The development of affinity among students. Journal of Experimental Social Psychology.

[20] Brockner J, Swap WC (1976). Effects of repeated exposure and attitudinal similarity on self-disclosure and interpersonal attraction. Journal of Personality and Social Psychology.

[21] Tajfel H (1970). Experiments in intergroup discrimination. Sci. Am..

[22] Sailer K, McCulloh I (2012). Social networks and spatial configuration—how office layouts drive social interaction. Social networks.

[23] Doreian P, Conti N (2012). Social context, spatial structure and social network structure. Social networks.

[24] Zajonc RB (1968). Attitudinal effects of mere exposure. Journal of Personality and Social Psychology.

[25] Davies K, Tropp LR, Aron A, Pettigrew TF, Wright SC (2011). Cross-Group Friendships and Intergroup Attitudes: A Meta-Analytic Review. Personality and Social Psychology Review.

[26] Pettigrew TF (1997). Generalized intergroup contact effects on prejudice. Personality and Social Psychology Bulletin.

[27] Wright SC, Aron A, McLaughlin-Volpe T, Ropp SA (1997). The extended contact effect: Knowledge of cross-group friendships and prejudice. Journal of Personality and Social Psychology.

[28] Brown, R. & Hewstone, M. *An integrative theory of intergroup contact*, vol. 37 (Academic Press, 2005).

[29] Pettigrew TF, Tropp LR (2008). How does intergroup contact reduce prejudice? Meta-analytic tests of three mediators. European Journal of Social Psychology.

[30] Stark, T. H. *Integration in schools: A process perspective on students’ interethnic attitudes and interpersonal relationships* (Rijksuniversiteit Groningen, 2011).

[31] Robins, G. *Doing social network research: Network-based research design for social scientists* (Sage, 2015).

[32] Veenstra R, Dijkstra JK, Steglich C, Van Zalk MH (2013). Network-behavior dynamics. Journal of Research on Adolescence.

[33] McPherson JM, Smith-Lovin L, Cook JM (2001). Birds of a feather: Homophily in social networks. Annual Review of Sociology.

[34] Dijkstra JK, Berger C, Lindenberg S (2011). Do physical and relational aggression explain adolescents’ friendship selection? the competing roles of network characteristics, gender, and social status. Aggressive Behavior.

[35] Poulin F, Pedersen S (2007). Developmental changes in gender composition of friendship networks in adolescent girls and boys. Developmental Psychology.

[36] Faris R, Felmlee D (2011). Status struggles: Network centrality and gender segregation in same-and cross-gender aggression. American Sociological Review.

[37] Rivera MT, Soderstrom SB, Uzzi B (2010). Dynamics of dyads in social networks: Assortative, relational, and proximity mechanisms. ASnnual Review of Sociology.

[38] Heider F (1946). Attitudes and cognitive organization. The Journal of psychology.

[39] Davis JA (1967). Clustering and structural balance in graphs. Human relations.

[40] Grund TU, Densley JA (2015). Ethnic homophily and triad closure: Mapping internal gang structure using exponential random graph models. Journal of Contemporary Criminal Justice.

[41] Stadtfeld C, Pentland A (2015). Partnership ties shape friendship networks: A dynamic social network study. Social Forces.

[42] Stadtfeld, C. The micro-macro link in social networks. *Emerging Trends in the Social and Behavioral Sciences* (2018).

[43] Feld SL (1981). The focused organization of social ties. American journal of sociology.

[44] Emerson RM (1976). Social exchange theory. Annual review of sociology.

[45] Merton RK (1968). The matthew effect in science. Science.

[46] Barabási A-L, Albert R (1999). Emergence of scaling in random networks. science.

[47] Baumeister RF, Leary MR (1995). The need to belong: desire for interpersonal attachments as a fundamental human motivation. Psychological bulletin.

[48] Kadushin, C. *Understanding social networks: Theories, concepts, and findings* (Oup Usa, 2012).

[49] Gould RV (2002). The origins of status hierarchies: A formal theory and empirical test. American Journal of Sociology.

[50] Burt, R. S. *Structural holes: The social structure of competition* (Harvard University Press, 2009).

[51] Allport, G.*W. The nature of prejudice*. (Wesley, 1954).

[52] Tropp, L. R. & Mallett, R. K. *Moving beyond prejudice reduction: Pathways to positive intergroup relations*. (American Psychological Association, 2011).

[53] Etzkowitz, H., Kemelgor, C. & Uzzi, B. *Athena unbound: The advancement of women in science and technology* (Cambridge University Press, 2000).

[54] Herzig AH (2004). Becoming mathematicians: Women and students of color choosing and leaving doctoral mathematics. Review of Educational Research.

[55] Best KL, Sanwald U, Ihsen S, Ittel A (2013). Gender and STEM in Germany: Policies Enhancing Women’s Participation in Academia. International Journal of Gender, Science and Technology.

[56] Isphording, I & Qendrai, P Gender Differences in Student Dropout in STEM. *IZA Research Report* **87**, (2019).

[57] Christopher SA, Elliott R, Adair R, Matier M, Scott J (1994). Choosing and leaving science in highly selective institutions. Research in Higher Education.

[58] Barbu S, Maner-Idrissi G, Jouanjean A (2000). The Emergence of Gender Segregation: Towards an Integrative Perspective. Current Psychology Letters. Behaviour, Brain & Cognition.

[59] Settle IH, Cortina LM, Malley J, Stewart AJ (2006). The climate for women in academic science: The good, the bad, and the changeable. Psychology of Women Quarterly.

[60] Goble P, Martin CL, Hanish LD, Fabes RA (2012). Children’s Gender-Typed Activity Choices Across Preschool Social Contexts. Sex Roles.

[61] Martin CL (2013). The Role of Sex of Peers and Gender-Typed Activities in Young Children’s Peer Affiliative Networks: A Longitudinal Analysis of Selection and Influence. Child Development.

[62] Halpern DF (2011). The Pseudoscience of Single-Sex Schooling. Science.

[63] Valente TW (2012). Network interventions. Science.

[64] Snijders TAB (2017). Stochastic actor-oriented models for network dynamics. Annual Review of Statistics and Its Application.

[65] Stadtfeld, C., Snijders, T. A. B., Steglich, C. & van Duijn, M. A. J. Statistical power in longitudinal network studies. *Sociological Methods & Research* (2018). Forthcoming.

[66] Etzkowitz H, Kemelgor C, Neuschatz M, Uzzi B, Alonzo J (1994). The paradox of critical mass for women in science. Science.

[67] Moody J (2001). Race, school integration, and friendship segregation in america. American journal of Sociology.

[68] R Core Team. *R: A Language and Environment for Statistical Computing*. R Foundation for Statistical Computing, Vienna, Austria (2013).

[69] Krackhardt D (1988). Predicting with networks: Nonparametric multiple regression analysis of dyadic data. Social Networks.

[70] Butts CT (2008). Social network analysis: A methodological introduction. Asian Journal of Social Psychology.

[71] Snijders TAB (2001). The statistical evaluation of social network dynamics. Sociological Methodology.

[72] Block, P., Stadtfeld, C. & Snijders, T. A. B. Forms of dependence: Comparing saoms and ergms from basic principles. *Sociological Methods and Research* (2016).

[73] Ripley, R. M., Snijders, T. A. B., Boda, Z. & Vörös, A. *Manual for SIENA 4.0*. Nuffield College and Department of Statistics, University of Oxford (2017). Version: March 29, 2019.

