# Supplementary material for: Short-term and long-term effects of a social network intervention on friendships among university students
Source: Sci Rep. 2020 Feb 19;10:2889. doi: 10.1038/s41598-020-59594-z (PMC7031228; doi:10.1038/s41598-020-59594-z)
Supplement: Supplementary file 1 — Supplementary Information. [file 41598_2020_59594_MOESM1_ESM.pdf]

Supplementary material for Short-term and long-term effects of a social network intervention on friendships among university students

Zsófia Boda[1,*,+], Timon Elmer[1,+], András Vörös[1,2,+], and Christoph Stadtfeld[1,,+]**

[1]Chair of Social Networks, Department of Humanities, Social and Political Sciences, ETH Zürich, Weinbergstrasse 109, Zürich, 8006, Switzerland
[2]Department of Social Statistics and Mitchell Centre for Social Network Analysis, University of Manchester, Oxford Road, Manchester, M13 9PL, United Kingdom
[*]zsofia.boda@gess.ethz.ch
[**]christoph.stadtfeld@ethz.ch
[+]these authors contributed equally to this work

ABSTRACT

This supplementary material provides (1) additional information on the broader context of the empirical study, (2) more details on friendship nominations, (3) complete results of and (4) robustness checks for the stochastic actor-oriented models, (5) comparisons of intervention participants and non-participants, (6) a post-hoc comparison of the friendship ties of students who participated and who did not participate at the Student Information Days (SIDs), and (7) robustness analyses on the randomization thresholds.

The Swiss StudentLife Study

Our data stem from the Swiss Student Life Study [SSL Study; 1], a longitudinal social network panel that surveyed a cohort of 226 students starting their studies at a competitive Swiss university in the 2016-2017 academic year. The main goal of the study was to understand the process of social integration within the cohort and how this affects the academic performance, study motivation, and mental health of students. Students in the sample moved to the university from various places in Switzerland and all around the world and hardly ever knew each other before. In the course of one year, however, they developed a densely-knit friendship network. After 12 months, they called on average 4.2 fellow students their friends. This network became in their lives: after a year, 61% of respondents answered that one of their best friends in life was from the cohort, and 78% had a friend among their fellow students whom they could turn to for emotional or study-related support. At the end of summer 2017, the students had to take a complex exam which determined whether they could continue their studies in the program. The exam was highly competitive, with only 48% of the students passing. Those who failed the exam could not continue their studies with their peers (though they had a chance to attend the exam one more time during the next academic year).

The studied cohort consisted of 222 individuals at the start of the study program. Some of the students (N = 46) decided to quit while others (N = 4) joined the cohort during the first year. The total sample size of 226 students takes into account everyone who was part of the cohort at the time of any survey waves. 81 (36%) of these students were female. The mean age of students was 20.00 years (SD = 2.03) at the beginning of the year. 47 students (21%) did not provide their age. Most individuals were born in Switzerland (50%), 20% were born in German-speaking countries, 10% were from the rest of the world, and 20% did not provide information on their birth country. 98 students (43%) had at least one parent with a university degree. 48 students (21%) did not provide information on their parents' education. Each student in the sample was admitted into one of the four study programs of the department with 97, 39, 59 and 31 students per program. Those in the same study program shared most of their classes during the year, but they also had ample opportunity to interact with peers from the other study programs. The whole cohort attended several classes together. During the academic year, the cohort size ranged between 222 students in the first wave and 180 students in the last wave. The number of students who freshly joined the cohort between each two waves (waves 1 and 2, waves 2 and 3, waves 3 and 4, waves 4 and 5, and waves 5 and 6) are 1, 0, 3, 0, and 0, respectively. The number of students who dropped out are 7, 3, 22, 0, and 14, respectively.

The response rate ranged from 77% in wave 1 to 61% in wave 6. The response rates are conservative estimates, as we

Table 1. Full results of the stochastic actor-oriented model for the creation of initial ties. The effects presented in Figure 5 of the main text are highlighted in the table.

Effect	Parameter	(S.E.)
Endogenous structural processes		
Outdegree	–5.252***	(0.679)
**Reciprocity**	4.436***	(0.390)
**Transitive triplets**	2.569***	(0.310)
Transitive reciprocated triplets	–2.206***	(0.541)
**Indegree-related popularity**	–0.154	(0.098)
Outdegree-related popularity	–0.617***	(0.160)
Outdegree-related activity	0.094**	(0.036)
Student Introduction Day		
**Same SID group**	0.639**	(0.234)
**Friend of same-SID-group member**	–0.001	(0.350)
**Same-SID-group member of friend**	–0.012	(0.155)
**Different gender in same SID group**	0.216	(0.275)
SID participation: alter	0.039	(0.168)
SID participation: ego	–0.570**	(0.180)
SID participation: both	–0.065	(0.122)
SID participation: same day	0.545*	(0.219)
Background characteristics		
Gender: alter	0.250†	(0.132)
Gender: ego	–0.110	(0.171)
**Gender: same**	0.324***	(0.098)
Country of origin: same	0.485***	(0.135)
**Study program A: both**	0.366**	(0.114)
**Study program B: both**	0.220	(0.141)
**Study program C: both**	0.355**	(0.124)
**Study program D: both**	0.420*	(0.196)
Mother's education: alter	0.105	(0.088)
Mother's education: ego	–0.011	(0.055)
Mother's education: same	0.052	(0.118)

All convergence t ratios < 0.03; overall maximum convergence ratio $= 0.19$
† $p < 0.1$; * $p < 0.05$; ** $p < 0.01$; *** $p < 0.001$

consider each student who is enrolled at the time of a survey a potential participant. We know, however, that there can be considerable gaps between the decision to leave the study program and the formal act of signing out.

The study followed a strict informed-consent policy that was reviewed and approved by the ethics board of ETH Zürich (ethics approval number: 2016-N-27).

Friendship nominations

The main variables of interest for this article were the friendship nominations of the students across the six survey waves. Plots of the friendship network of the cohort in the waves that are not displayed in Figure 1 of the main text (waves 2, 3, 4, and 6) are shown in Figure 1 here. Node colors indicate the Student Introduction Day (SID) grouping. Friendship ties within the SID groups are colored according to the group, other ties are gray. For the description of the SID groups, see the Methods section of the main text.

Students had the opportunity to nominate up to 20 peers in the friendship network. Figure 2 presents the outdegree distribution of students in each wave, that is, how many peers they nominated as friends. The red line represents the average number of nominations in the given wave. The maximum observed outdegree is 15 (in wave 5), which suggests that the upper limit provided for friendship nominations probably did not influence the results.

To assess the stability of the friendship network over time, we calculate a Jaccard-index for each period. This measure shows the proportion of friendship ties that are present at both the beginning and the end of the period, compared to all ties present either at the beginning or the end. These are 0.24, 0.42, 0.58, 0.59, and 0.65 in the consecutive periods, showing that friendships become more stable relationships over time. It is important to note that all of these values are ideal for fitting stochastic actor-oriented models [2].

Full results of the stochastic actor-oriented models

Table 1 presents the full SAOM specification and results, which are summarized in Figure 5A of the main text. In the model, the dependent variable is whether an individual (ego) creates or maintains a friendship tie towards another individual (alter). We

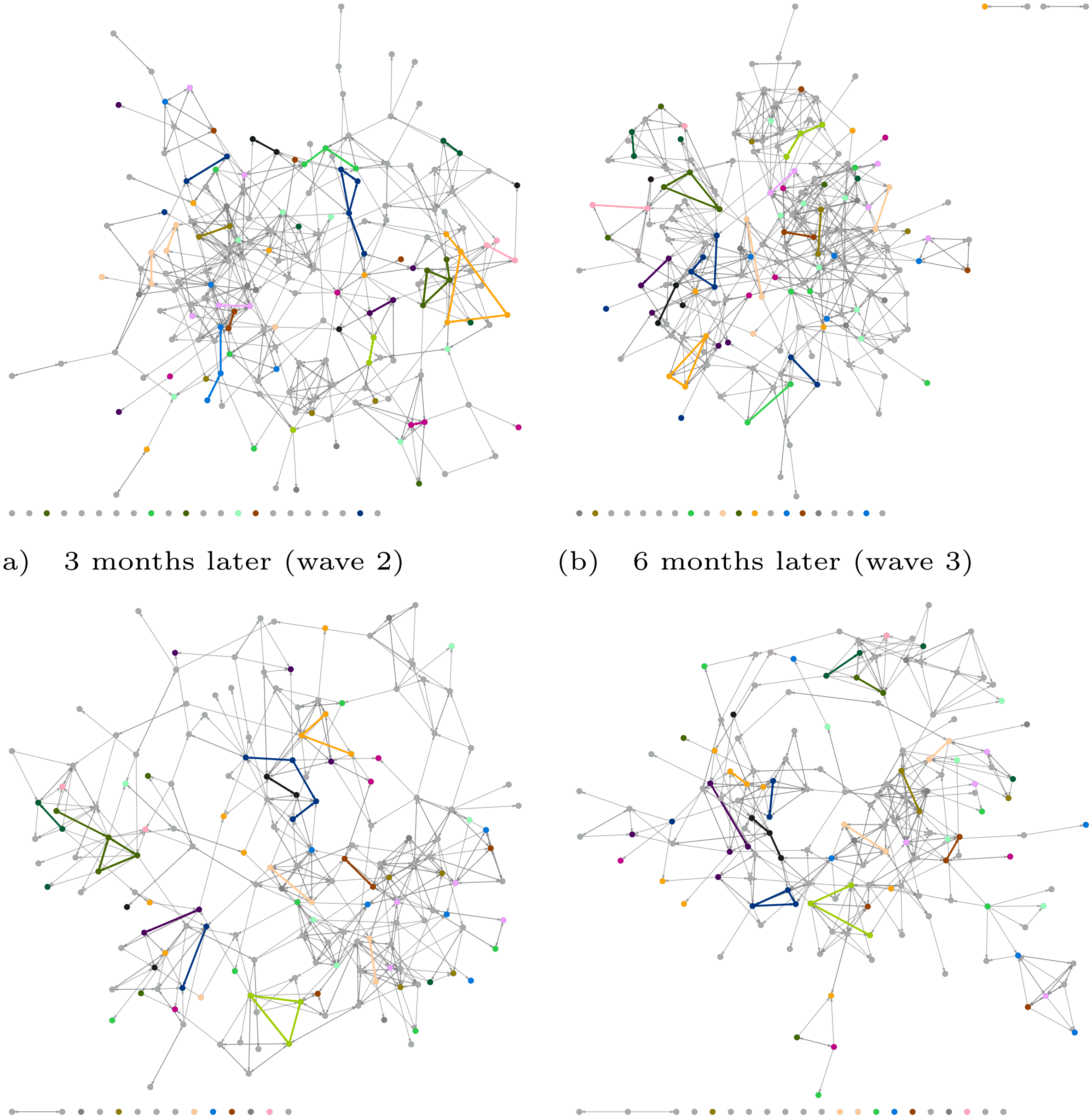

(a) 3 months later (wave 2)

(b) 6 months later (wave 3)

(c) 9 months later (wave 4)

(d) 9 months later (wave 5)

Figure 1. The friendship networks of the cohort at waves 2, 3, 4, and 5. Friendship ties that coincide within-group ties are highlighted in the respective color, others are shown in gray. Waves 1 and 6 are presented in Figure 1 of the main text.

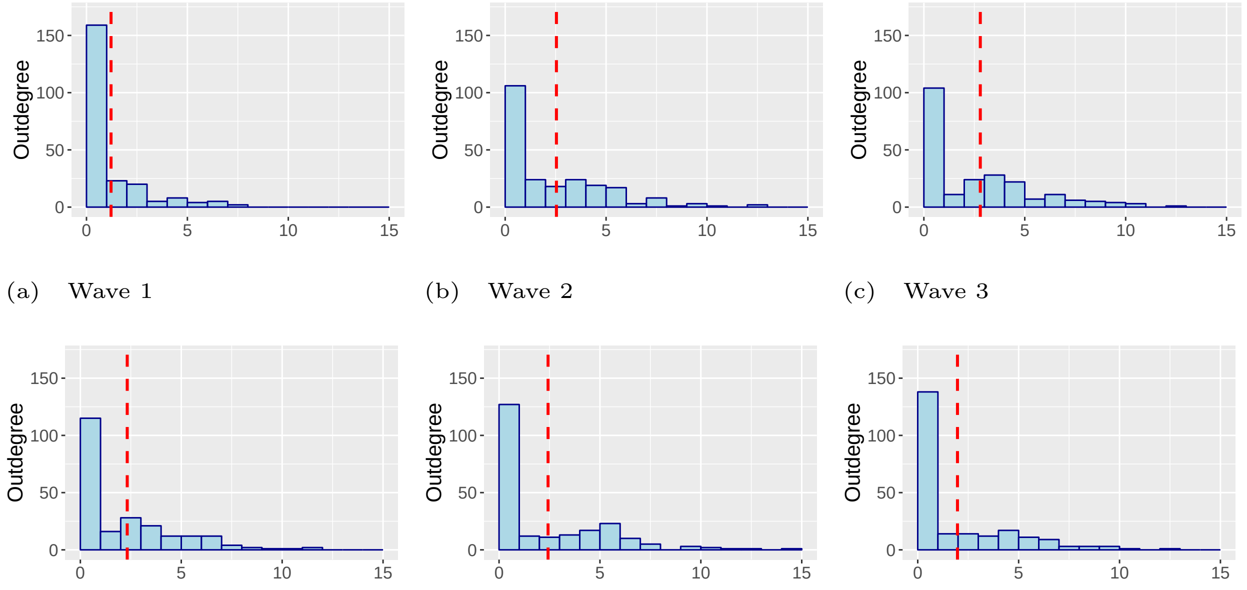

(a) Wave 1 (b) Wave 2 (c) Wave 3

(d) Wave 4 (e) Wave 5 (f) Wave 6

Figure 2. Friendship outdegree distribution in the six waves. The red line shows the mean outdegree of students in the given wave.

start the simulation process from an empty network, and use the first observation of friendship as the target network. The rate parameter captures the extent of change that happens between each pair of subsequent waves, while the rest of the parameters express effects of various independent variables. In this model, the rate parameter was not estimated but fixed to 20, allowing an average individual to make 20 changes in her outgoing ties. This was done in order to achieve a converged model. As a robustness check, the model was also estimated with a rate parameter of 50, with similar results.

The rest of the parameters in the model capture the effects of three groups of independent variables on friendship ties. In terms of endogenous structural processes, the negative outdegree parameter shows that everything else being equal, individuals are less likely to be connected than not connected with each other. This variable serves as an intercept in the model. Reciprocity is positive and significant: friendship ties tend to become and remain reciprocated. The transitive triplets parameter is also positive and significant, signaling that individuals tend to form and maintain transitive structures. The transitive reciprocated triplets parameter is, at the same time, negative and significant, capturing an interaction between reciprocity and transitivity. This effect has been interpreted as showing that transitivity is less important for friendship ties when reciprocity is present (and vice versa) [3]. Finally, there are three degree-related effects in the model. The first one captures preferential attachment processes and is negative though non-significant. Therefore, in this early phase of the network formation we find no evidence on the self-reinforcing nature of popularity. The second one refers to the tendency of those with higher outdegrees to be nominated more by others; this is negative and significant. The third one, showing whether those with higher outdegrees tend to increase/maintain the number of their outgoing ties is positive and significant.

The SID variables show whether those attending the SID, and those being sorted into the same group, are different in their social ties than others. The same SID group variable is negative, which means that conditioning on the observed friendship network in one wave (which is highly affected by the network intervention, see Figure 2 in the main text), ties between those from the same SID group are less likely to be created or maintained than ties between others. The effect is positive and significant. As described in the main text, SID-related closure effects are not significant in the model. The nomination of different-gender peers within the same SID group is positive and relatively large, but not significant. SID participation itself also has some effect on early friendship. Those who participated are not nominated more or less than others, but they themselves nominate less, given all other variables in the model. Students who both participated do not nominate each other more than other students. Those who participated on the same day, though, are significantly more likely to name each other as friends later. Interpreting these results jointly, the SID participation ego and the same-day participation variables are similar in absolute

Table 2. Full results of the stochastic actor-oriented model for network evolution between waves 1 and 6. The effects presented in Figure 5 of the main text are highlighted in the table.

**Effect**	**Parameter**	**(S.E.)**
Rate parameters		
Wave 1 → 2	13.692	(1.40)
Wave 2 → 3	9.054	(0.80)
Wave 3 → 4	3.479	(0.29)
Wave 4 → 5	4.250	(0.36)
Wave 5 → 6	2.770	(0.26)
Endogenous structural processes		
Outdegree	–5.112***	(0.31)
**Reciprocity**	3.479***	(0.15)
**Transitive triplets**	0.976***	(0.05)
Transitive reciprocated triplets	–0.712***	(0.07)
**Indegree-related popularity**	0.079***	(0.02)
Outdegree-related popularity	–0.244***	(0.02)
Outdegree-related activity	–0.005	(0.01)
Student Introduction Day		
**Same SID group**	–0.307†	(0.18)
**Friend of same-SID-group member**	0.078	(0.09)
**Same-SID-group member of friend**	0.029	(0.05)
**Different gender in same SID group**	0.259	(0.24)
SID participation: alter	0.082	(0.06)
SID participation: ego	–0.134†	(0.07)
SID participation: both	–0.001	(0.05)
SID participation: same day	0.059	(0.10)
Background characteristics		
Gender: alter	0.230***	(0.05)
Gender: ego	–0.069	(0.07)
**Gender: same**	0.193***	(0.05)
Country of origin: same	0.101	(0.07)
**Study program A: both**	0.340***	(0.06)
**Study program B: both**	0.258***	(0.07)
**Study program C: both**	0.410***	(0.06)
**Study program D: both**	0.897***	(0.12)
Mother's education: alter	0.093**	(0.03)
Mother's education: ego	0.017	(0.03)
Mother's education: same	–0.113	(0.07)

All convergence t ratios < 0.04; overall maximum convergence ratio $= 0.11$
† $p < 0.1$; * $p < 0.05$; ** $p < 0.01$; *** $p < 0.001$

size, showing that those who participated at a SID are unlikely to name those as friends who were not at their SID day, while the most likely is nomination between those who were in the same group (same SID group + participation ego + same-day participation).

In terms of background characteristics, we find that female students are more popular friends than male students in general, while individuals are also likely to make gender-homophilous friendship choices. We also find homophily in country of origin and though, in study program—students in three of the four programs are significantly more likely to name each other as friends than peers who study in different programs (in study program B, we find a similar tendency but it is not significant). Finally, we find no significant effect of parental education.

Table 2 presents the full SAOM specification and results, which are summarized in Figure 5B of the main text. Again, in the model the dependent variable is whether an individual (ego) creates or maintains a friendship tie towards another individual (alter). The rate parameters express the extent of change that happens between each pair of subsequent waves, while the rest of the parameters express effects of various independent variables.

Rate parameters are estimated separately for each time period of observed network change. We have six waves of data, therefore, we have five time periods. Each rate parameter expresses the number of chances an average actor gets to change her outgoing ties during the observed period. These changes are included in a simulation model that is at the core of the SAOM estimation routine [2]. Results show that in the first period (between waves 1 and 2), actors change their ties a lot. In the second period (between waves 2 and 3), the amount of change decreases. In the third period (between waves 3 and 4), there is a further decrease, but afterwards, it remains more or less the same. This finding is in line with the descriptive Figure 4C in the .

Like in the previous model, the rest of the parameters in the model capture the effects of three groups of independent variables on friendship ties. We find similar endogenous structural processes as in the model exploring initial friendship

evolution: the outdegree is negative and significant, the reciprocity band transitive triplet parameters are positive and significant. We again see a negative and significant effect for transitive reciprocated triplets. Results for the degree-related effects, are, however, somewhat different. We now find evidence for preferential attachment processes. This confirms our notion on the self-reinforcing nature of popularity (although note that this does not necessarily mean that popularity differences between students increase over time). Outdegree-related popularity, referring to the tendency of those with higher outdegrees to be nominated more by others, is now negative and significant. The outdegree-related activity variable is again non-significant.

Results on the SID variables are very different from those in the model capturing initial network evolution. The same SID group variable is negative, which means that conditioning on the observed friendship network in one wave (which is highly affected by the network intervention, see Figure 2 in the main text), ties between those from the same SID group are less likely to be created or maintained than ties between others. The effect is significant only on the 10% level, however. SID-related closure effects are again non-significant, and same-gender ties in the same SID group are not significantly more likely to be created or maintained than other ties (though the effect is again relatively large). SID participation also does not have a strong effect on friendships, though we have evidence at the $p < 0.1$ significance level that those who attended nominate fewer friends in general (again, net of all other effects and conditioning on the wave 1 observation in which SID participants reported more ties). In terms of background characteristics, we again find evidence for female students being more popular friends than male students in general, and for gender homophily. We do not find evidence for homophily in country of origin anymore. There is strong homophily, though, in study program—students in all four programs are more likely to name each other as friends than peers who study in different programs. Finally, we find that those with higher parental education are somewhat more likely to be named as friends.

Robustness checks for friendship evolution

This section contains three additional analyses using SAOMs: first, we present a period-wise model for friendship evolution; second, we control for the size of one's SID group; third, we split the four main SID effects into two effects each in order to assess the creation of new ties and stability of existing ties separately.

First, the main analysis assumed time homogeneity of parameters across waves; here we estimate the same model for each of the five time periods separately. Results are presented in Table 3. In general, we see similar tendencies to those found in our main model, though fewer parameters are significant in each period than in the overall model. This may be due to decreased statistical power. In terms of SID effects in particular, being in the same group is negative in three periods (two of which are significant), and positive in two (none of which are significant). We do not observe any clear patterns behind these sign changes. Further, we find non-significant positive and negative effects for becoming or staying friends with friends of same-SID-group members, or with same-SID-group members of friends (though in the latter case, two periods show effects that are significant on the 0.1 level – one of these is positive, while the other one is negative). Finally, same-gender ties in the same SID group are first (non-significantly) less likely, then (non-significantly) more likely to be created or maintained than other ties (though this latter tendency is marginally significant in the fourth period).

Second, it is possible that the size of the SID group one was sorted into affects how much this person is connected to others in the same group later. While SAOMs take opportunity structure (e.g. proportion of same-SID members and others in the cohort) into account, it may be true that the same mechanisms work differently given this opportunity structure. We therefore include a variable in our main model that captures the SID-group size of individuals who attended the SID (that is, the number of SID participants who actually enrolled at the university later), and interact this with our four main SID effects in the model. Results for the initial period are presented in Table 4, and results for the friendship evolution between the first and the sixth wave are in Table 5. Based on Table 4, in the initial period students are significantly more likely to befriend those they were in the same SID group with, but there is no significant difference in this based on SID-group size. The rest of the SID effects are not significant, and are also not significantly different depending SID-group size. Based on Table 5, students are not significantly influenced by SID-related variables in the rest of the academic year; though there is a difference that is significant on the 10% level in the level of creation and maintenance of SID-group ties depending on group size: the non-significant negative effect of SID membership is larger in absolute value if the SID group had more members.

Finally, same-SID group membership may affect the formation of new ties and the stability of existing ties differently. For instance, while we may not expect individuals to form new ties to old same-SID peers late in the academic year, it is plausible that their existing ties will be more stable. We test this in an additional SAOM, in which we separate our four main SID effects into creation and stability effects [2]. Table 6 shows significant positive parameters both for becoming friends with friends of same-SID-group members, and with same-SID-group members of friends. At the same time, we see significant negative parameters for the stability of both of these nomination types. Therefore, these types of relationships are significantly more likely to be created than other ties, but are also significantly more likely to dissolve over time.

Table 3. Period-wise models. Results of five separate period-wise stochastic actor-oriented models for network evolution between waves 1 and 6.

	Wave 1 → 2		Wave 2 → 3		Wave 3 → 4		Wave 4 → 5		Wave 5 → 6	
**Effect**	**Parameter**	**(S.E.)**	**Parameter**	**(S.E.)**	**Parameter**	**(S.E.)**	**Parameter**	**(S.E.)**	**Parameter**	**(S.E.)**
Rate	6.697	(0.433)	8.1317	(0.658)	3.418	(0.278)	4.041	(0.335)	2.509	(0.222)
Endogenous structural processes										
Outdegree	-5.674***	(0.674)	-5.468***	(0.518)	-5.008***	(0.852)	-5.071***	(0.834)	-6.916***	(1.563)
**Reciprocity**	3.936***	(0.387)	3.312***	(0.235)	3.022***	(0.362)	3.212***	(0.374)	3.810***	(0.531)
**Transitive triplets**	1.438***	(0.209)	0.947***	(0.089)	0.834***	(0.115)	1.040***	(0.096)	1.437***	(0.180)
Transitive reciprocated triplets	-0.669	(0.456)	-0.758***	(0.134)	-0.466**	(0.175)	-0.720***	(0.138)	-1.066***	(0.219)
**Indegree-related popularity**	0.079*	(0.035)	0.061*	(0.025)	0.026	(0.050)	0.111*	(0.048)	0.089	(0.066)
Outdegree-related popularity	-0.160**	(0.049)	-0.213***	(0.037)	-0.198***	(0.052)	-0.271***	(0.051)	-0.324***	(0.095)
Outdegree-related activity	0.027	(0.022)	-0.008	(0.012)	0.010	(0.019)	0.017	(0.017)	-0.051†	(0.029)
Student Introduction Day										
**Same SID group**	-0.227	(0.414)	-0.852*	(0.423)	0.474	(0.555)	-1.342*	(0.626)	0.346	(0.940)
**Friend of same-SID-group member**	-0.290	(0.335)	0.104	(0.165)	0.160	(0.240)	0.178	(0.224)	-0.137	(0.373)
**Same-SID-group member of friend**	-0.153	(0.159)	0.122†	(0.071)	-0.257†	(0.146)	-0.004	(0.107)	-0.041	(0.172)
**Different gender in same SID group**	-0.014	(0.579)	0.346	(0.547)	0.134	(0.680)	1.442†	(0.840)	0.105	(1.134)
SID participation: alter	0.198	(0.138)	0.059	(0.120)	0.273†	(0.162)	-0.068	(0.152)	0.155	(0.254)
SID participation: ego	0.032	(0.185)	-0.122	(0.146)	-0.050	(0.197)	-0.154	(0.183)	-0.422	(0.346)
SID participation: both	0.033	(0.118)	-0.060	(0.105)	0.310†	(0.175)	-0.269†	(0.151)	0.221	(0.239)
SID participation: same day	0.073	(0.218)	-0.189	(0.193)	-0.142	(0.285)	0.246	(0.299)	-0.133	(0.494)
Background characteristics										
Gender: alter	0.244†	(0.130)	0.400***	(0.097)	-0.044	(0.189)	0.235*	(0.128)	0.216	(0.254)
Gender: ego	-0.314	(0.204)	0.147	(0.124)	0.161	(0.181)	-0.134	(0.166)	0.032	(0.285)
**Gender: same**	0.182†	(0.107)	0.154	(0.099)	0.365*	(0.179)	0.085	(0.147)	0.546*	(0.228)
Country of origin: same	-0.152	(0.136)	0.173	(0.117)	0.143	(0.190)	-0.108	(0.148)	0.500†	(0.284)
**Study program A: both**	0.368*	(0.143)	0.269*	(0.120)	0.559**	(0.194)	0.457*	(0.207)	0.472†	(0.287)
**Study program B: both**	0.157	(0.141)	0.424**	(0.146)	0.459†	(0.235)	0.297	(0.186)	0.233	(0.317)
**Study program C: both**	0.471***	(0.134)	0.543***	(0.121)	0.393†	(0.237)	0.325†	(0.174)	0.249	(0.294)
**Study program D: both**	1.351***	(0.275)	0.952***	(0.240)	0.412	(0.266)	1.097***	(0.331)	1.400**	(0.539)
Mother's education: alter	0.125*	(0.059)	-0.018	(0.051)	0.126	(0.100)	-0.001	(0.071)	0.257	(0.179)
Mother's education: ego	0.038	(0.057)	0.059	(0.044)	-0.120	(0.082)	0.097	(0.075)	0.024	(0.153)
Mother's education: same	-0.249	(0.157)	0.045	(0.128)	-0.260	(0.216)	-0.090	(0.168)	-0.707†	(0.366)

All convergence t ratios < 0.09; overall maximum convergence ratio $= 0.25$

† $p < 0.1$; * $p < 0.05$; ** $p < 0.01$; *** $p < 0.001$

Table 4. Model with SID-group size for the creation of initial ties. Results of the stochastic actor-oriented model for the creation of initial ties, taking SID-group size into account.

**Effect**	**Parameter**	**(S.E.)**
Endogenous structural processes		
Outdegree	–5.284***	(0.729)
**Reciprocity**	4.428***	(0.494)
**Transitive triplets**	2.557***	(0.269)
Transitive reciprocated triplets	–2.221***	(0.662)
**Indegree-related popularity**	–0.148	(0.110)
Outdegree-related popularity	–0.601**	(0.184)
Outdegree-related activity	0.097**	(0.033)
Student Introduction Day		
**Same SID group**	0.625*	(0.251)
**Same SID group × SID-group size**	–0.192	(0.170)
**Friend of same-SID-group member**	–0.025	(0.408)
**Friend of same-SID-group member × SID-group size**	0.275	(0.212)
**Same-SID-group member of friend**	–0.028	(0.162)
**Same-SID-group member of friend × SID-group size**	0.050	(0.270)
**Different gender in same SID group**	0.196	(0.290)
**Different gender in same SID group × SID-group size**	0.048	(0.138)
SID participation: alter	0.037	(0.196)
SID participation: ego	–0.556**	(0.195)
SID participation: both	–0.068	(0.127)
SID participation: same day	0.545*	(0.242)
SID-group size	–0.053	(0.088)
Background characteristics		
Gender: alter	0.245†	(0.133)
Gender: ego	–0.097	(0.154)
**Gender: same**	0.327***	(0.096)
Country of origin: same	0.489***	(0.132)
**Study program A: both**	0.367***	(0.109)
**Study program B: both**	0.228†	(0.138)
**Study program C: both**	0.354**	(0.116)
**Study program D: both**	0.406*	(0.161)
Mother’s education: alter	0.107	(0.083)
Mother’s education: ego	–0.016	(0.054)
Mother’s education: same	0.055	(0.113)

All convergence t ratios < 0.1; overall maximum convergence ratio $= 0.23$

† $p < 0.1$; * $p < 0.05$; ** $p < 0.01$; *** $p < 0.001$

Table 5. Model with SID-group size for network evolution between waves 1 and 6. Results of the stochastic actor-oriented model for network evolution between waves 1 and 6, taking SID group size into account.

**Effect**	**Parameter**	**(S.E.)**
Rate parameters		
Wave 1 $\rightarrow$ 2	13.552	(1.432)
Wave 2 $\rightarrow$ 3	9.055	(0.744)
Wave 3 $\rightarrow$ 4	3.443	(0.278)
Wave 4 $\rightarrow$ 5	4.259	(0.362)
Wave 5 $\rightarrow$ 6	2.781	(0.255)
Endogenous structural processes		
Outdegree	–5.101***	(0.270)
**Reciprocity**	3.486***	(0.147)
**Transitive triplets**	0.978***	(0.048)
Transitive reciprocated triplets	–0.714***	(0.071)
**Indegree-related popularity**	0.080***	(0.017)
Outdegree-related popularity	–0.243***	(0.028)
Outdegree-related activity	–0.006	(0.008)
Student Introduction Day		
**Same SID group**	–0.293	(0.190)
**Same SID group $\times$ SID-group size**	–0.307†	(0.18)
**Friend of same-SID-group member**	–0.033	(0.068)
**Friend of same-SID-group member $\times$ SID-group size**	0.078	(0.09)
**Same-SID-group member of friend**	0.011	(0.036)
**Same-SID-group member of friend $\times$ SID-group size**	0.029	(0.05)
**Different gender in same SID group**	–0.104	(0.191)
**Different gender in same SID group $\times$ SID-group size**	0.259	(0.24)
SID participation: alter	0.088	(0.061)
SID participation: ego	–0.124†	(0.071)
SID participation: both	0.006	(0.053)
SID participation: same day	0.046	(0.102)
SID-group size	0.078†	(0.040)
Background characteristics		
Gender: alter	0.231***	(0.053)
Gender: ego	–0.085	(0.073)
**Gender: same**	0.190***	(0.048)
Country of origin: same	0.097	(0.060)
**Study program A: both**	0.340***	(0.065)
**Study program B: both**	0.246***	(0.071)
**Study program C: both**	0.423***	(0.073)
**Study program D: both**	0.877***	(0.112)
Mother's education: alter	0.091**	(0.032)
Mother's education: ego	0.023	(0.030)
Mother's education: same	–0.112†	(0.064)

All convergence t ratios < 0.07; overall maximum convergence ratio $= 0.24$

† $p < 0.1$; * $p < 0.05$; ** $p < 0.01$; *** $p < 0.001$

Table 6. Model for creation and stability of ties. Results of the stochastic actor-oriented model for network evolution between waves 1 and 6, separating SID effects into separate parameters for creation of new ties and stability of existing ties.

**Effect**	**Parameter**	**(S.E.)**
Rate parameters		
Wave 1 → 2	12.818	(1.306)
Wave 2 → 3	8.832	(0.732)
Wave 3 → 4	3.439	(0.279)
Wave 4 → 5	4.183	(0.352)
Wave 5 → 6	2.740	(0.244)
Endogenous structural processes		
Outdegree	–5.080***	(0.301)
**Reciprocity**	3.507***	(0.137)
**Transitive triplets**	0.987***	(0.052)
Transitive reciprocated triplets	–0.711***	(0.076)
**Indegree-related popularity**	0.075**	(0.025)
Outdegree-related popularity	–0.246***	(0.029)
Outdegree-related activity	–0.002	(0.008)
Student Introduction Day		
**Same SID group, creation**	–0.335	(0.313)
**Same SID group, stability**	–0.204	(0.467)
**Friend of same-SID-group member, creation**	0.662***	(0.196)
**Friend of same-SID-group member, stability**	–0.818**	(0.289)
**Same-SID-group member of friend, creation**	0.361***	(0.077)
**Same-SID-group member of friend, stability**	–0.602***	(0.139)
**Different gender in same SID group, creation**	–0.046	(0.454)
**Different gender in same SID group, stability**	0.655	(0.542)
SID participation: alter	0.071	(0.062)
SID participation: ego	–0.100	(0.081)
SID participation: both	–0.006	(0.054)
SID participation: same day	0.076	(0.100)
Background characteristics		
Gender: alter	0.232***	(0.053)
Gender: ego	–0.073	(0.070)
**Gender: same**	0.189***	(0.053)
Country of origin: same	0.107†	(0.061)
**Study program A: both**	0.331***	(0.064)
**Study program B: both**	0.260***	(0.071)
**Study program C: both**	0.408***	(0.068)
**Study program D: both**	0.879***	(0.123)
Mother's education: alter	0.097**	(0.033)
Mother's education: ego	0.016	(0.027)
Mother's education: same	–0.119†	(0.068)

All convergence t ratios < 0.12; overall maximum convergence ratio $= 0.24$

† $p < 0.1$; * $p < 0.05$; ** $p < 0.01$; *** $p < 0.001$;

Table 7. Individual differences between intervention participants and non-participants. T-tests and chi-squared tests examining whether intervention participants differ from non-participants along certain characteristics.

Dimension	Participant mean	Non-participant mean	p
Openness	3.50	3.76	0.069
Conscientiousness	3.44	3.27	0.193
Extraversion	3.08	3.04	0.764
Agreeableness	3.26	3.33	0.603
Neuroticism	2.99	2.73	0.092
Age	19.96	20.00	0.885
**Dimension**	**Participant proportion**	**Non-participant proportion**	**p**
Female	38%	34 %	0.573
Swiss nationality	89%	80 %	0.135

Results of t-tests (for continuous variables) and Chi-squared tests (for binary variables)

Do intervention participants differ from those who did not participate?

The participants of this study could choose themselves whether they want to participate in one of the two SIDs and thus become part of the intervention (i.e., a quasi-experimental design). Therefore, those who participated in the intervention might differ along some characteristics from those that did not participate. For this reason, we conducted additional analyses where we tested whether participants differed from non-participants with regard to socio-demographic characteristics (age, gender, and Swiss nationality) and the Big Five personality traits (measured in wave 1 with the BFI-10 [4]). The p values from t-tests (for continuous personality and age variables) and chi-squared tests (for binary gender and swiss nationality variables) are reported in Table 7. The analyses indicate that the intervention participants did not significantly differ from the non-participants along these dimensions.

Friendships of intervention participants and non-participants

In a post-hoc analysis, we compare the characteristics of the friendships of students who participated and who did not participate at the SIDs. This serves only to assess descriptively whether there are differences between the two groups of students in the statistics used for Figures 2 and 3 in the main text. Due to self-selection to participate at the events, e.g. based on country of origin and potentially sociability, these comparisons cannot be used to evaluate the effects of our intervention, they only complement the main analyses. However, we find this useful and necessary, because within our core experimental setting of randomized SID groups (which is in the focus of the main text), we can only compare the realized mixed-gender ties within SID groups to mixed-gender ties between students who attended the same SID event (see the Methods section of the main text for more details). Individuals without exposure to the intervention, who did not participate at the SIDs, do not appear in that comparison.

The average number of friendships, that of two-step friendships and the proportion of mixed-gender friendships of SID participants (upper blue lines) are compared to those of non-participants (lower red lines) in Figure 3. Through time, those who did not participate in the SID have a consistently lower values on each of the three statistics. Further, by applying a multiple regression method developed for network data, MRQAP [5], we can test whether the tendency of SID participants to form mixed-gender ties is higher than that of non-participants, *controlling for* the general tendency to create friendships and the general tendency to have mixed-gender friendships. In the model, this test is expressed as an interaction effect, where the two main effects are the tendency of SID participants to have more friends than non-participants do (estimates are between -0.02 and 0.12 and non-significant in all waves), and the general tendency of students to have mixed-gender friends (estimates are between -0.63 and -0.29 and significant in all waves, indicating gender homophily). The interaction effect is consistently positive (between 0.04 and 0.25; as expected under an effective intervention) but non-significant in the six waves (p-values: 0.18, 0.40, 0.14, 0.34, 0.16, 0.15). We get the same result if we add such an interaction effect to the stochastic actor-oriented model in Table 2 (p-value: 0.10).

These results complement our main findings and point out that the intervention might have been successful in terms of fostering social integration in general and mixed-gender friendships in particular. However, these descriptive findings have to be taken with a grain of salt as the setting is not truly experimental. SID participation was not random and the two groups differ in terms of sociability or other unmeasured factors that may be correlated with social integration. Future research should investigate the link between mixed-gender contact opportunities and friendships in more detail, potentially applying rigorous experimental research designs similar to those in the main analyses of this study.

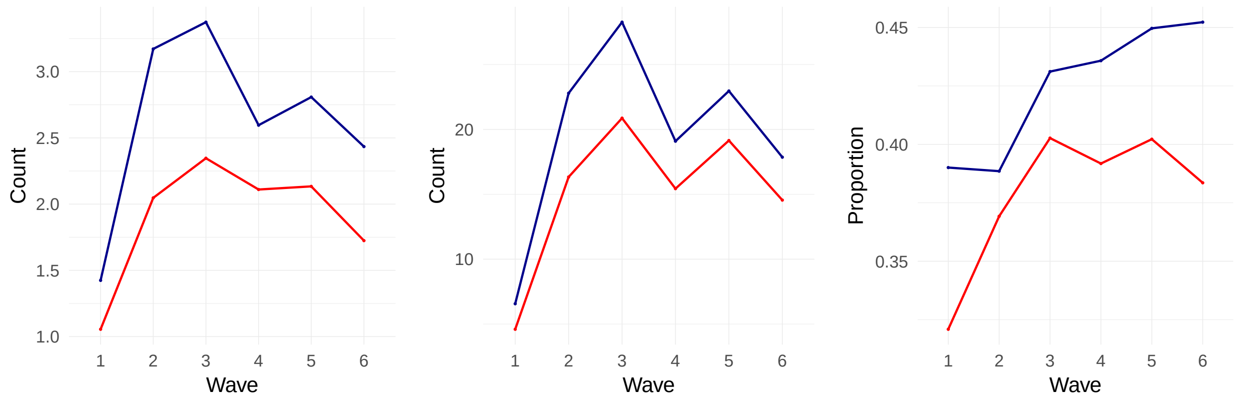

(a) Direct friendship (b) Two-step friendship (c) Mixed-gender friendship

Figure 3. Differences in friendship ties between SID participants and non-participants. Average number of direct friendships, two-step friendships and average proportion of mixed-gender friendships of SID participants (higher blue lines) and non-participants (lower red lines).

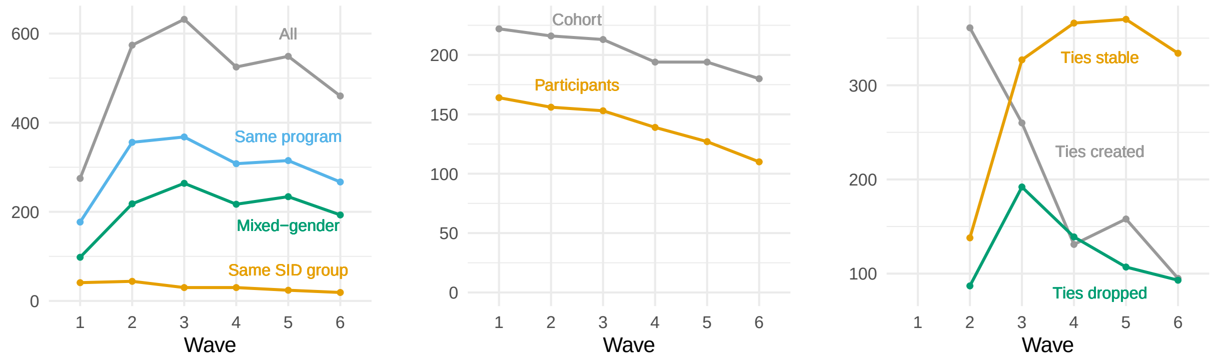

(a) Number of ties (b) Number of individuals (c) Network change

Figure 4. Replication with alternative gender-ratio thresholds. Replication of results with null-distributions from hypothetical SID groupings with gender-ratio thresholds of 0.25 (a-c), 0.30 (d-f; the actual threshold), 0.30 (g-i) and 0.50 (j-l).

Alternative randomization thresholds for group assignment

The results reported and shown in Figures 2 and 3 in the main text describe certain characteristics of the friendships ties between students who were assigned to the same SID group (same SID group friendships). The considered characteristics are expressed by three statistics: the number of same SID group friendships, the number two-step friendships (two-paths or shared friends) connecting students in the same SID group (regardless of whether they are friends themselves), and the number of same SID group friendships between students of the opposite gender (mixed-gender ties).

In a non-parametric test, which is akin to permutation tests often applied to network data, each of the three observed statistics is compared against a null-distribution calculated from hypothetical SID groupings. The hypothetical groupings represent different assignments of students to SID groups that could have resulted from our randomization procedure. This way, we are able to assess if the observed characteristics of friendships between students in the same SID group are expected by chance given the randomization algorithm. We do this for each of the three statistics in each of the six survey waves.

To ensure we compare the statistics against the right null-distributions, we used a the same algorithm and gender-ratio threshold as in the original randomized assignment of students to SID groups when generating the hypothetical groupings. The algorithm is formally presented in the Methods section of the main text.

The gender-ratio threshold allowed a maximum of 0.3 female ratio difference between the SID groups of the same study program and the same SID day. SID groupings that showed a larger heterogeneity between groups were discarded and a new grouping was generated until it fell under the threshold. As a result, the expected gender ratio in each group coming from our algorithm was closer to the marginal ratio than in case we used a simple uniform random assignment algorithm.

The gender-ratio threshold was never considered a variable but chosen as constant in the randomization and in the generation of hypothetical groupings for the analyses in the main text. However, the actual randomization could have been realized under different thresholds. Therefore, we report further robustness checks with alternative gender-ratio thresholds of 0.25, 0.35 and 0.50. Figure 4 shows that using alternative thresholds results in almost identical patterns regarding the effect size and significance of the reported results of the main text.

References

1. Stadtfeld, C., Vörös, A., Elmer, T., Boda, Z. & Raabe, I. J. Integration in emerging social networks explains academic failure and success. *Proc. Natl. Acad. Sci.* **116**, 792–797 (2019).

2. Ripley, R. M., Snijders, T. A. B., Boda, Z., Vörös, A. & Preciado Lopez, P. *Manual for SIENA 4.0.* Nuffield College and Department of Statistics, University of Oxford (2017). Version: March 29, 2019 (with effects added by the authors).

3. Block, P. Reciprocity, transitivity, and the mysterious three-cycle. *Soc. Networks* **40**, 163–173 (2015).

4. Rammstedt, B. & John, O. P. Measuring personality in one minute or less: A 10-item short version of the big five inventory in english and german. *J. research Pers.* **41**, 203–212 (2007).

5. Dekker, D., Krackhardt, D. & Snijders, T. A. B. Sensitivity of mrqap tests to collimearity and autocorrelation conditions. *Psychometrika* **72**, 563–581 (2007).
